# Whole genome sequence analysis and characterization of virulent Newcastle disease virus isolates from chicken and pheasants during 2020/21 outbreaks in Nepal

**DOI:** 10.1186/s12985-025-03035-8

**Published:** 2025-12-07

**Authors:** Meera Prajapati, Aashirbad Pokharel, Shresha Rayamajhi, Madhav Prasad Acharya, Manita Aryal, Suruchi Karn, Joe James, Ashley C. Banyard, Craig S. Ross, Joshua G. Lynton-Jenkins

**Affiliations:** 1 National Animal Health Research Center, Nepal Agricultural Research Council, Khumaltar, Lalitpur, Nepal; 2 Himalayan College of Agricultural Sciences and Technology, Kirtipur, Kathmandu, Nepal; 3 Central Department of Microbiology, Tribhuvan University, Kirtipur, Kathmandu, Nepal; 4 Department of Virology, Animal and Plant Health Agency (APHA- Weybridge), Woodham Lane, Addlestone, Surrey, KT15 3NB UK; 5 WOAH/FAO International Reference Laboratory for Avian Influenza, Animal and Plant Health Agency (APHA-Weybridge), Woodham Lane, Addlestone, Surrey, KT15 3NB UK

**Keywords:** Whole genome, Newcastle disease, F protein, Molecular epidemiology

## Abstract

Newcastle Disease (ND) remains a major threat to poultry production worldwide, particularly in regions where it is endemic, like Southern Asia. The disease is caused by virulent forms of avian paramyxovirus-1, commonly termed Newcastle Disease Virus (NDV), a highly contagious virus with significant genetic diversity and evolving pathogenicity. This study aimed to molecularly characterize NDV isolates obtained from chickens and pheasants during the 2020/21 ND outbreaks in Nepal, to understand their genetic makeup, phylogenetic relationships, and implications for control strategies. Necropsy samples, including trachea, liver, intestine, spleen, lungs, heart, and proventriculus were collected from ten birds. Isolates from five clinical samples were typed as NDV by hemagglutination and hemagglutination inhibition (HA/HI) assays and were subjected to whole genome sequencing (WGS). Full genomes of 15,192 nucleotides were recovered from each isolate. Fusion (F) gene sequence analysis revealed the presence of multi-basic cleavage site motif ^112^RRQKRF^117^ in all isolates, indicative of virulent strain and suggesting a potentially velogenic or mesogenic phenotype. Phylogenetic analyses consistently classified all isolates within genotype VII.2 of class II NDV. Further comparative analysis indicated a close genetic relationship between the Nepalese isolates and strains reported from India and Bangladesh, and BEAST analysis suggested Southern Asia as the likely source of introduction into Nepal. These viral genomes provide additional insight into contemporary NDV circulating in an area of endemicity.

## Introduction

Newcastle disease (ND), caused by virulent forms of avian paramyxovirus-1 (APMV-1), commonly termed Newcastle Disease Virus (NDV), is a highly contagious, devastating viral infection of domesticated and wild birds. The infection can cause severe clinical disease, high mortality, and significant economic losses due to decreased productivity, increased mortality, and associated trade restrictions. ND remains endemic in many parts of the world, particularly in resource limited settings, where outbreaks lead to high flock mortalities and threaten poultry-based livelihoods.

The causative agent, avian paramyxovirus-1, is an enveloped, single stranded negative sense RNA virus belonging to the family *Paramyxoviridae*, genus *Orthoavulavirus* [46]. The viral genome is approximately 15.2 kilobases in length and encodes six structural proteins: nucleocapsid protein (NP), phosphoprotein (P), matrix protein (M), fusion protein (F), haemagglutinin-neuraminidase (HN), and large RNA-dependent polymerase (L) in the order of 3’-N-P-M-F-HN-L-5’ [9]. Two accessory proteins are encoded from the P gene following ribosomal slippage, namely V and W [51]. NDV strains are broadly classified into four pathotypes: avirulent, lentogenic (low morbidity, no mortality), mesogenic (high morbidity, low mortality), and velogenic (high morbidity and high mortality), with velogenic strains causing either viscerotropic disease (with intestinal haemorrhaging) or neurotropic disease (with neurological symptoms such as torticollis and encephalitis). A key molecular marker of virulence is the fusion (F) protein cleavage site (CS). All virulent forms have a multi-basic cleavage site from positions 113–116 and a phenylalanine at position 117, allowing cleavage of the F protein from inactive F_0_ to the active form F_1_ and F_2_ by Furin-like proteases [37, 57], ubiquitously expressed in multiple cell types, thus resulting in systemic infection, including detection in the brain [2]. Cleavage sites of low or avirulent viruses have di-basic sequence at position 113–116 and a leucine at position 117, cleaved by trypsin-like proteases which are only detected in the respiratory and intestinal tracts, and thus limits infections to these tissues [8, 16]. The presence of a virulent CS is one of the determinants for declaring ND in a poultry flock, along with a score of > 0.7 from the diagnostic Intracerebral Pathogenicity Index (ICPI) assay, as decreed by the World Organisation for Animal Health (WOAH). Besides these molecular markers, antigenic epitopes on the F and HN proteins, the viral surface glycoproteins, play a crucial role in host immune recognition and vaccine efficacy. Several neutralizing epitopes have been mapped on the F protein, while the HN protein contains major antigenic sites that mediate receptor binding and neuraminidase activity, making both genes key targets for molecular characterization and comparative analysis [19, 36].

Although all APMV-1 are of a single serotype, phylogenetic analysis of the F gene has resulted in the distinction of two APMV-1 classes (class I and II) [11]. Class I viruses are largely avirulent in nature and mostly found in wild birds [11] whereas class II contains twenty genotypes (I – XXI, with genotype XV unassigned) of both avirulent strains (e.g. vaccine strains La Sota, Hitchner B1, Ulster 2C), and virulent strains responsible for the majority of ND outbreaks worldwide [11, 30]. The severity and outcome of ND outbreaks are largely determined by the virulence of the circulating NDV strain and its interaction with the host immune response and vaccination status. The use of vaccines is common in both countries where NDV is absent and endemic [27]. Despite global vaccination efforts, NDV continues to cause outbreaks which could be due to improper vaccine storage, handling, and administration, as well as incomplete flock coverage [11, 13, 23].

APMV-1 has been detected in over 241 different bird species, in 27 different orders [20]. Infections with velogenic NDV strains are most commonly reported in domesticated Galliformes, whereas lentogenic and avirulent variants are also detected in wild and aquatic birds [11, 28]. Studies have indicated that Phasianidae (e.g., pheasants and partridges), are equally susceptible to virulent ND genotype VII as chickens and, where raised in backyard settings, and have the potential to serve as intermediaries between wild birds and domestic poultry [1, 48]. However, where pheasants are more typically reared in commercial settings, such as in South Asia, reports of pheasant-specific outbreaks are rare.

Nepal is a resource-limited country in South Asia where poultry farming plays a vital role in food security and income generation, particularly among rural populations. The poultry industry comprises a mixture of commercial and backyard systems, both of which are vulnerable to infectious diseases such as ND. Although vaccination is widely practiced (primarily using genotype II strains), ND outbreaks remain frequent and severe, undermining disease control efforts [33, 42]. NDV outbreaks have been reported in pheasants and chickens [38, 42]. More recent outbreaks from 2021 were identified as genotype VII.2 and published partial sequencing of the fusion protein gene [33, 42]. However, few studies have investigated the molecular characteristics of these emerging strains in Nepal.

Genotype VII of NDV was initially identified in the Far East during the mid-1980s [19, 25], with the first known isolate later determined to have occurred in Indonesia in 1976 [31] and has since emerged as a major cause of ND outbreaks across Southeast, East, and South Asia, as well as parts of Africa and Europe [17]. This genotype has become endemic in many countries and is associated with large-scale poultry losses and economic disruption [7, 12]. Although genotype VII strains are increasingly detected in Nepal, there is a lack of whole genome sequencing (WGS) data to characterize their genetic makeup. Without detailed molecular information, it is difficult to track virus evolution, identify markers of virulence, or transmission [47]. Following an outbreak in poultry in the Kathmandu region of Nepal in 2020/21, NDV was identified and isolated from chickens and pheasants. We performed WGS of five NDV isolates and analysed them phylogenetically to understand their genetic features, virulence determinants, and evolutionary relationships. This is the first study to apply WGS to NDV strains circulating in Nepal and is expected to generate crucial insights for guiding future vaccine development and improving national disease control strategies.

## Methodology

### Sample collection and processing

Poultry suspected of NDV infection were brought for postmortem examination (PME) at the National Animal Health Research Center (NAHRC), Khumaltar, Lalitpur. Organ samples, including trachea, liver, intestine, spleen, lungs, heart and proventriculus were collected at the time of necropsy. The lesions observed during necropsy were haemorrhages in trachea, gastrointestinal tract, pinpoint haemorrhages in proventriculus, nephritis, hepatomegaly and splenomegaly. Samples from birds which showed the lesion commonly associated with ND virus and detected positive by NDV antigen test (Bionote, Cat no: RG1503DD) were stored at − 80^o^ C. Altogether, 18 organ samples from ten birds were collected for virus isolation from pheasants and backyard chickens (Table 1). These samples were promptly processed for virus isolation at NAHRC, Nepal and NDV positive allantoic fluids were kept at − 80 °C until it was sent to Animal and Plant Health Agency, UK for whole genome sequencing.

**Table 1:** Summary of clinical samples used for virus isolation including sampling location and host species

Sample ID	Sampling date	Poultry	Location	Tissue pool	Isolate HA titre	NCBI Accession
1	10th August 2020	Backyard chicken	Lalitpur	Trachea, spleen	2^7^	PQ408134
2	10th August 2020	Backyard chicken	Lalitpur	Trachea, spleen	2^11^	PQ655419
3	5-9th August 2020	Backyard chicken	Kathmandu and Lalitpur	Proventriculus (pool of four chickens)	2^8^	PQ655420
4	7th March 2021	Backyard chicken	Lalitpur	(Pooled of Intestine, proventriculus, liver, spleen and trachea)	2^6^	PQ655422
5	4th October 2021	Pheasant	Bhaktapur	Brain, spleen, proventriculus, crop	2^9^	PQ655421

### Virus isolation

For each bird, multiple tissue samples were collected, pooled and a 20% (w/v) tissue homogenate was prepared using phosphate buffered saline supplemented with gentamycin (10 mg/ml). The suspension was placed at 4^o^ C for 2 h and then clarified by centrifugation at 3000 rpm for 10 min. Approximately 0.2 ml of the supernatant from the tissue homogenate was inoculated into the allantoic cavity of 10 days old embryonated fowls eggs (EFEs) which were tested as NDV free as stated by WOAH [59]. The eggs were incubated at 37 °C until the embryos died or for a maximum period of 96 h. Embryos were candled every 24 h, and the dead embryos were stored at 4 °C prior to collection of the allantoic fluids. Embryos that died before 24 h of incubation were excluded. The harvested allantoic fluid from each egg was checked for hemagglutination activity (HA) [59]. HA positive allantoic fluids were confirmed as NDV by haemagglutination inhibition test (HI) using the ND antiserum (Ulster Newcastle Disease Virus Antiserum, UK. Lot no: 1/20) at NAHRC, Nepal and by rRT-PCR at the Animal and Plant Health Agency (APHA), UK.

### RNA Extraction, PCR and detection

Nucleic acid extraction was performed using the QIAmp viral RNA mini kit (QIAGEN, Manchester, UK) according to the manufacturer’s instructions. The presence of APMV-1 viral RNA (vRNA) in tissues was determined using the L-gene rRT-PCR assay [43, 53].

### Whole genome sequencing and phylogenetic analysis

WGS of five virus isolates from the incursion was carried out as described previously [43]. In brief, cDNA was generated using the SuperScript IV First-Strand Synthesis System with random hexamers (ThermoFisher), and then to double-stranded cDNA using the NEBNext Ultra II Non-Directional RNA Second Strand Synthesis Module (New England Biolabs). cDNA was purified using Agencourt AMPure XP beads (Beckman Coulter). Subsequently, 1 ng of purified dsDNA was used as template in the sequencing library generated using the Nextera XT kit (Illumina). Sequencing was performed on a NextSeq 550 (Illumina) with 2 × 150 base paired end reads. Raw sequencing reads were assembled using a custom de novo assembly approach. F gene sequences were combined with representative APMV-1 sequences obtained from GenBank (www.ncbi.nlm.nih.gov/genbank/) and from the NDV consortium sequence database (https://github.com/NDVconsortium/NDV_Sequence_Datasets). Sequences were first aligned using Mafft [22], ModelFinder [21] was then used to determine an appropriate phylogenetic model and trees were inferred using maximum-likelihood models in IQ-Tree v2.2.5 [29] with 1,000 ultrafast bootstraps [18].

Bayesian phylogenetic analysis of selected full F-gene sequences was carried out using BEAST v 1.1.10.4 [52] in combination with Beagle library [4]. We employed an uncorrelated relaxed clock and constant population size (see Table S1) using a General Time Reversible substitution model [55] with separate partitions for codon positions 1 plus 2 versus position 3. Two independent MCMC chains with a length of 200,000,000 and sampling every 20,000 iterations were carried out, with the first 10% discarded as the burn-in. Convergence was assessed using Tracer v1.7.2 [39]. The maximum clade credibility (MCC) tree was summarized using TreeAnnotator v1.10.4 [52] and visualized using R with the tidyverse, treeio and phytools packages [45, 56, 58]. To determine regional spread, APMV-1 samples were designated a region determined by the UN geoscheme [54] with the Nepal designated independently. To explore the pattern of spatial diffusion among the geographic regions, discrete phylogeographic analyses using location as a trait were performed [26]. We assumed an asymmetric non-reversible transition model and incorporated Bayesian stochastic search variable selection [26]. SpreaD3 was used to measure rates of transmission using Bayes Factor (BF) and was used to determine likelihood of transmission between locations [5]. The support for BF transmission was as described previously [24]. BF and representative transitions were visualized as described previously [50]. Markov jump counts were used to measure the number of viral movements along the branches of the phylogeny and estimated the Markov rewards to quantify the time the virus spent in each geographical region [32]. As an alternative method to examine evolutionary and geographic spread, analysis was carried out using the mugration model in Treetime, using default settings [49]. The analysis was carried out on a previously generated M-L tree, with molecular clock estimation, using the UN geoscheme designated regions.

For the analysis of amino acid mutations in the F and HN genes, the nucleotide sequences of these genes from all five isolates were translated into amino acid sequences using the translate function from the Biopython Seq module. Along with these, comparative sequences included the reference strain APMV 1/chicken/NL/152,608/93 (GenBank accession AEZ00711) and four vaccine strains: LaSota (AAC28374.1), Mukteswar (EF201805.1), Ulster 2 C (PQ301174.1) and Queensland V4 (JX524203.1). All sequences were aligned using MAFFT v7.505 with default parameters. The F gene cleavage site (residues 112–117) was analysed to determine the virulence-associated motif. Epitope regions in both the F and HN genes were identified based on previously reported studies and examined for strain-specific amino acid substitutions. Comparative analysis was performed using custom Python scripts to extract and tabulate mutations at the defined positions.

## Results

### Haemagglutination and haemagglutination inhibition test

Of the ten initial sample pools analysed, five isolation attempts were found negative in HA/HI testing and five were found positive for APMV-1 in HA/HI. A second passage was performed on the positive isolate and confirmed to be APMV-1 by rt-PCR analysis with WGS carried out on each sample. The details of the samples are shown in Table 1.

### Phylogenetic and comparative genome analysis

**Fig. 1:**
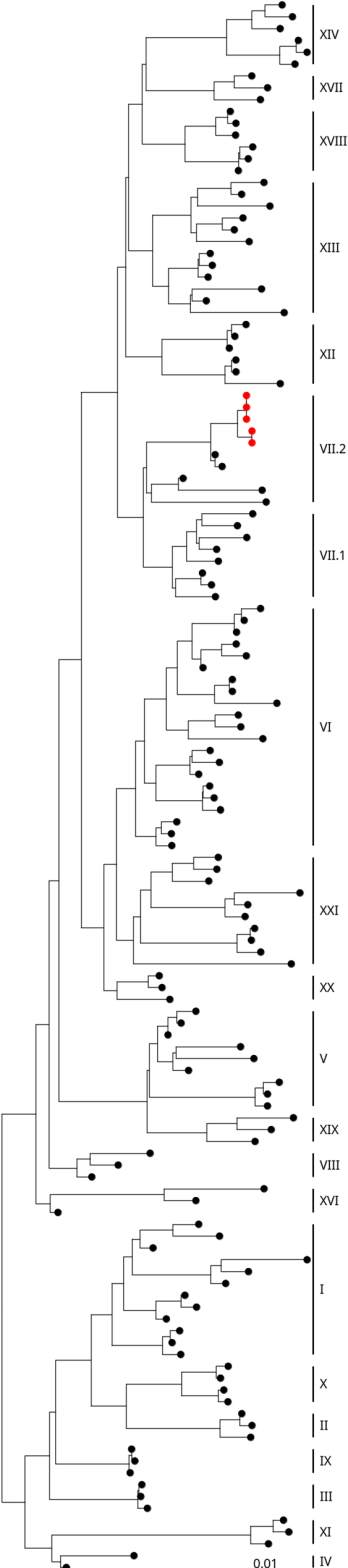
APMV-1 sequences obtained from Nepal are demonstrated to be genotype VII.2. Maximum-likelihood phylogenetic tree of the F-gene from global, pre-defined APMV-1 sequences. Sequences are coloured red for isolates from this study and black for all other sequences

Following WGS, the full F-gene sequence from across all twenty APMV-1 genotypes was used to determine the genotype of the five Nepalese isolates. Analysis demonstrated that all five Nepalese isolates were genotype VII.2 (Fig. 1 and Figure S1) as described by the classification system [11]. Three of the isolates clustered in one group with pairwise nucleotide identity analysis of the F gene showing them to be identical at the nucleotide level (100% identity), the remaining two isolates formed a second group also sharing 100% within-group F gene identity.

Further analysis of F-gene sequences previously designated as genotype VII.2 demonstrated that the Nepalese samples were closely related to samples observed in India and Bangladesh between 2020 and 2024 (Figure S2). For example, strains reported from Guwahati and Chhattisgarh in India showed the closest pairwise nucleotide identity with the two unique Nepalese F gene sequences reported in this study (NDV/Owl/Guwahati/01/20 (MZ546197) [15], 99.3% and 99% identity with Nepalese strains; NDV/Chicken/RPR/01/23 (OR185447) [41], 99.1% and 98.8% identity). While the next closest strains were reported from Chattogram in Bangladesh (e.g., APMV-1/NDV/Chicken/BD/37o/2024 (PV656753), 99.1% and 98.8% identity).

**Fig. 2:**
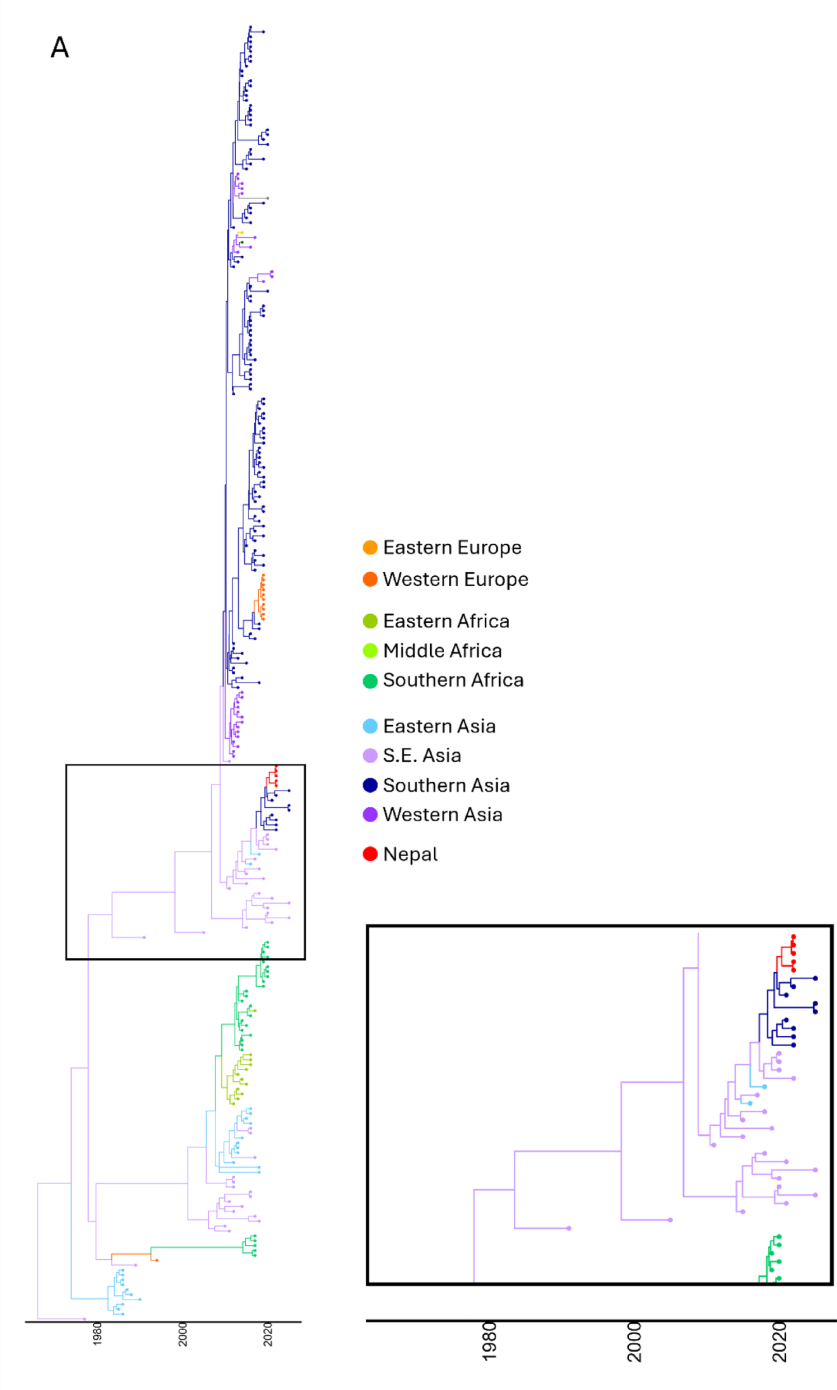

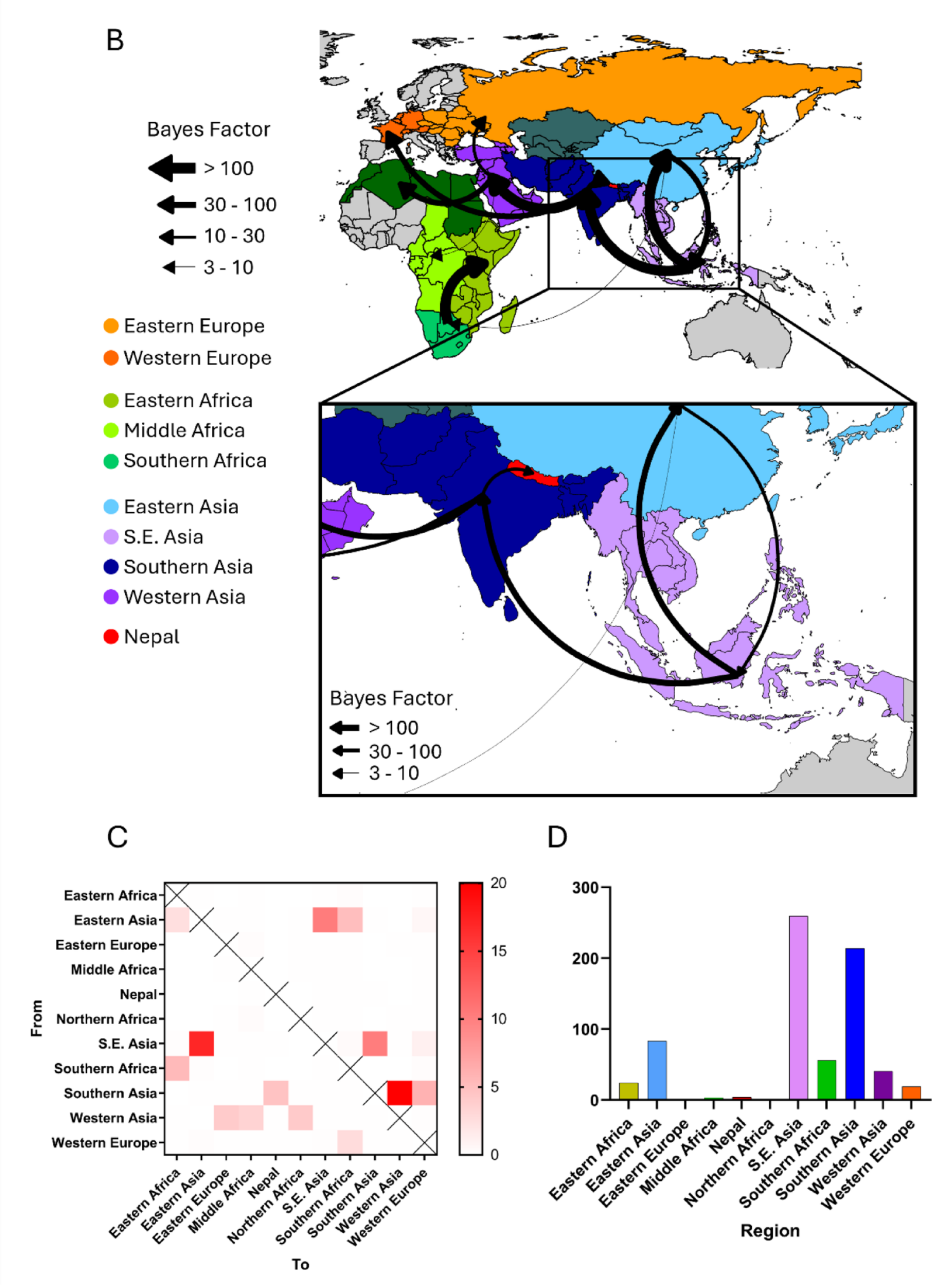
APMV-1 sequences designated genotype VII.2 are closely related to previous strains detected in South Asia. (**A**) MCC phylogeny of APMV-1 F-gene sequences previously identified as genotype VII.2 scaled to year of collection. Tips and branches are coloured by region of origin. (**B**) Analysis of APMV-1 spread between geographic regions. Regions with genotype VII.2 strains are coloured according to the UN geoscheme. Arrows indicate direction of transmission and thickness denotes strength of support. (**C**) Frequency of transitions for genotype VII.2 strains between regions determined by Markov jump counts. (**D**) Proportion of time genotype VII.2 viruses have been present in each region.

To further determine the incursion of virulent APMV-1 into Nepal, BEAST analysis was carried out. This determined that the most likely region of entry into Nepal was from Southern Asia (Fig. 2A, B and C) with the time to most recent common ancestor (TMRCA) to isolates from India estimated to be September 2018 (range Nov 2017 - Dec 2019, [Treetime Feb 2018, range Jan 2016 - Mar 2018] (Figure S3)). As previously noted, within Nepal, the five viruses split into two distinct clades, with the TMRCA for the Nepalese split predicted to be May 2019 (range June 2018 - Mar 2020, [Treetime-not determined]).

Spread analysis suggests that, with a high degree of posterior probability, that the ancestor was originally in South-east Asia, transitioned into Southern Asia, and subsequently moved into Nepal, when these incursions were detected (Fig. 2B and Table S2). This is backed up by the phylogenetic analysis where the last common ancestor prior to detection in Nepal was from Southern Asia (Fig. 2A and Figure S3). The TMRCA from South-east Asia into Southern Asia was estimated to have occurred in April 2016 (range Feb 2015 – May 2017, [Treetime Feb 2015, range June 2014 – Sept 2016]). Markov jump analysis suggests that the precursors transitioned from South-east Asia to both Southern and Eastern Asia, before transitioning from Southern Asia into Nepal (Fig. 2 C). Markov jump counts suggest that the precursors to the Nepalese incursion spent the longest time in South-east Asia, before being transmission into both Southern and Eastern Asia (Fig. 2D), and subsequently into Nepal (via Southern Asia).

### F and HN protein analysis

F gene cleavage site analysis showed a motif ¹¹²RRQKR/F¹¹⁷ in all five isolates. In contrast, the vaccine strains possessed either ¹¹²GRQGR/L¹¹⁷ or ¹¹²GKQGR/L¹¹⁷ motifs, except for the Mukteshwar strain, which showed ¹¹²RRQRR/F¹¹⁷. Mukteshwar, being mesogenic, is commonly used as a booster in ND endemic regions and was included for sequence comparison along with other vaccine strains in this study. Furthermore, amino acid analysis of the F and HN genes revealed no major substitutions or alterations in the field isolates when compared with the reference and vaccine strains (Table S3).

## Discussion

Though ND is endemic in Nepal, detailed study on the circulating virus is limited. This is the first study conducting whole genome sequencing with phylogeographic analysis of viral isolates from infected poultry, identifying genotype VII.2 of class II NDV as the causative agent of the outbreaks.

NDV strains belonging to genotype VII, which can be further categorized into genotype VII.1.1, VII.1.2 and VII.2 based on amino acid substitutions, are implicated in numerous outbreaks of virulent Newcastle disease (ND) across Asia, Africa, Europe and the Middle East since the late 1990 s [11, 12, 31]. Among these, genotype VII.2 has emerged as a dominant lineage, often associating with panzootic-like spread and high mortality in poultry across South-East and Southern Asia [11]. The findings from the whole genome sequencing revealed that all five samples analysed belong to genotype VII.2, concordant with previous findings [33] who also reported genotype VII.2 from NDV outbreaks in 2021. These five isolates clustered into two groups each with 100% within-group F gene identity. Two isolates forming one group were isolated from samples originating from two distinct farms (pheasants and chickens), whereas the second group of three isolates were collected from the same farm at different time points during the outbreak. The nucleotide identity between the two groups was 98.74%, indicating minor sequence differences between them. The identification of genotype VII.2 in Nepal underscores its persistence in the region [33, 42]. Spread analysis and Markov jump analysis demonstrated that the initial pre-cursor isolates were present in South-east Asia, before transitioning into Southern Asia (TMCRA estimation April 2016 [range Feb 2015 – May 2017]) before subsequently spreading throughout India, Bangladesh and Nepal. These South-east Asian isolates have also caused outbreaks in Pakistan, which were subsequently linked to incursions in Western Asia and Western Europe (Fig. 2B). This spread of Genotype VII.2 virus across Southern, South-eastern and Western regions of Asia demonstrate the difficulty of control of this virus, even where vaccination is routinely practised.

Genotype VII.2 is historically associated with velogenic strains of NDV, with high ICPI values resulting in severe systemic disease and high mortality in poultry. The conservation of the F-gene cleavage site (¹¹²RRQKR/F¹¹⁷) in all field isolates strongly suggests their virulent nature, aligning with class II virulent NDV strains known to cause outbreaks in poultry. This is consistent with previous studies reporting that multiple NDV outbreaks in Asia are driven by virulent strains with multi-basic cleavage sites [12]. Analysis of F gene revealed that the Nepalese isolates were closely related to India and Bangladesh between 2020 and 2023. Though directly not assessed in this study, this close genetic relationship may suggest potential cross-border viral movement influenced by regional poultry trade, migratory birds, or biosecurity lapses. In this study, three ND isolates were isolated from a vaccinated flock of 2–3 months old layers. Similarly, previous study reported infection of ND in vaccinated poultry [42]. Vaccination together with strict biosecurity measures is important to contain the disease. However, both biosafety and biosecurity measures were found to be generally poor in commercial chicken farms across Nepal [33]. This condition is worse in the case of backyard poultry where there is no biosecurity present. A pilot study reported that farms achieved only 42% compliance with personnel safety standards and 3% for rodent control, reflecting the widespread absence of disinfectants, footbaths, and protective clothing for staff [40]. In addition, field vaccination practices often suffer from inadequate cold chain maintenance, improper handling or reconstitution of vaccines, and lack of standardized administration schedules, all of which can reduce vaccine efficacy [6]. Moreover, feed contamination with mycotoxins such as aflatoxin B₁ has been reported in Nepalese poultry feed [3] and can impair immune responses, reducing antibody production even after proper vaccination [35]. These factors collectively contribute to reduced vaccine efficiency and recurrent NDV outbreaks despite routine vaccination.

Structural weaknesses such as substandard housing, free-ranging birds, and insecure feed storage further enabled access by wild birds and rodents, significantly elevating disease risk [10]. In such conditions, introduction and transmission of disease is easy from farm to farm contributing to the endemic nature of NDV in this region. The open border to India and proximity with Bangladesh, combined with the lack of strict veterinary checks, provides a critical pathway for the introduction of NDV into Nepal. Informal trade in live birds, hatching eggs, and poultry products from neighbouring Indian states, where repeated outbreaks of genotype VII have been documented, likely facilitated cross-border incursions of NDV [34]. In addition, Nepal’s position along the Central Asian Flyway, with shared wetlands and transboundary protected areas such as Chitwan–Valmiki, allows the movement of migratory birds and raptors between these countries. Although the precise role of wild birds in genotype VII transmission remains debated, their frequent interaction with backyard poultry in border regions may have further promoted viral spillover [14]. Once introduced, the weak biosecurity practices as described above may have allowed NDV to spread rapidly within Nepal, thereby establishing endemic circulation.

## Conclusions

This study has successfully isolated ND virus that circulated among poultry population in 2021 and provided whole genome sequences of NDV isolates offering valuable insights into molecular epidemiology. Phylogenetic analysis demonstrated close links with isolates from Bangladesh and India, with proposed routes of incursion outlined. The identification of genotype VII.2 strains in vaccinated birds demonstrates the capacity of this virus to cause outbreaks despite flock vaccination, highlighting existing limitations in local biosecurity and the application of effective vaccination.

## Supplementary Information

Supplementary Material 1

## Data Availability

Sequencing data available on NCBI (accession numbers: PQ408134, PQ655419, PQ655420, PQ655422 and PQ655421).
