# Supplementary material for: Whole genome sequence analysis and characterization of virulent Newcastle disease virus isolates from chicken and pheasants during 2020/21 outbreaks in Nepal
Source: Virol J. 2025 Dec 7;23:9. doi: 10.1186/s12985-025-03035-8 (PMC12798023; doi:10.1186/s12985-025-03035-8)
Supplement: Supplementary file 1 — Supplementary Material 1 [file 12985_2025_3035_MOESM1_ESM.docx]

**Supplementary Figure Legends**

**Supplementary figure 1.** APMV-1 sequences obtained from Nepal are demonstrated to be genotype VII.2. Maximum-likelihood phylogenetic tree of the F-gene from global, pre-defined APMV-1 sequences, with each sequence named. Sequences are coloured red for isolates from this study and black for all other sequences.

**Supplementary figure 2.** Maximum-likelihood analysis of APMV-1 sequences obtained from Nepal genotype VII.2 sequences. Maximum-likelihood phylogenetic tree of the F-gene from the Nepalese isolates and pre-defined genotype VII.2 isolates with each sequence named. Nepalese sequences from this study are coloured red, genotype VII.2 sequences are coloured black, and the root sequence is coloured orange.

**Supplementary figure 3.** APMV-1 sequences designated genotype VII.2 are closely related to previous strains detected in Southern Asia. Treetime analysis of APMV-1 F-gene sequences previously identified as genotype VII.2 scaled to year of collection. Tips and branches are coloured by region of origin.

**
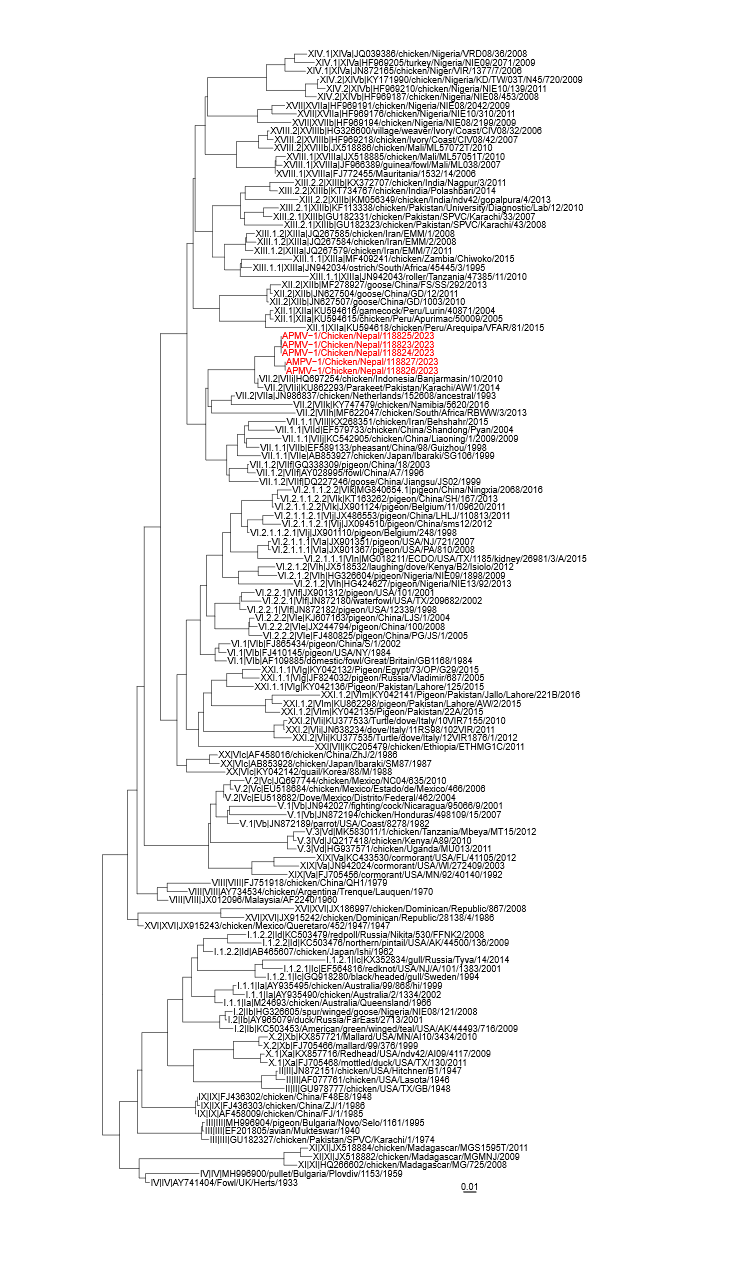
**

**Supplementary figure 1.** APMV-1 sequences obtained from Nepal are demonstrated to be genotype VII.2

**
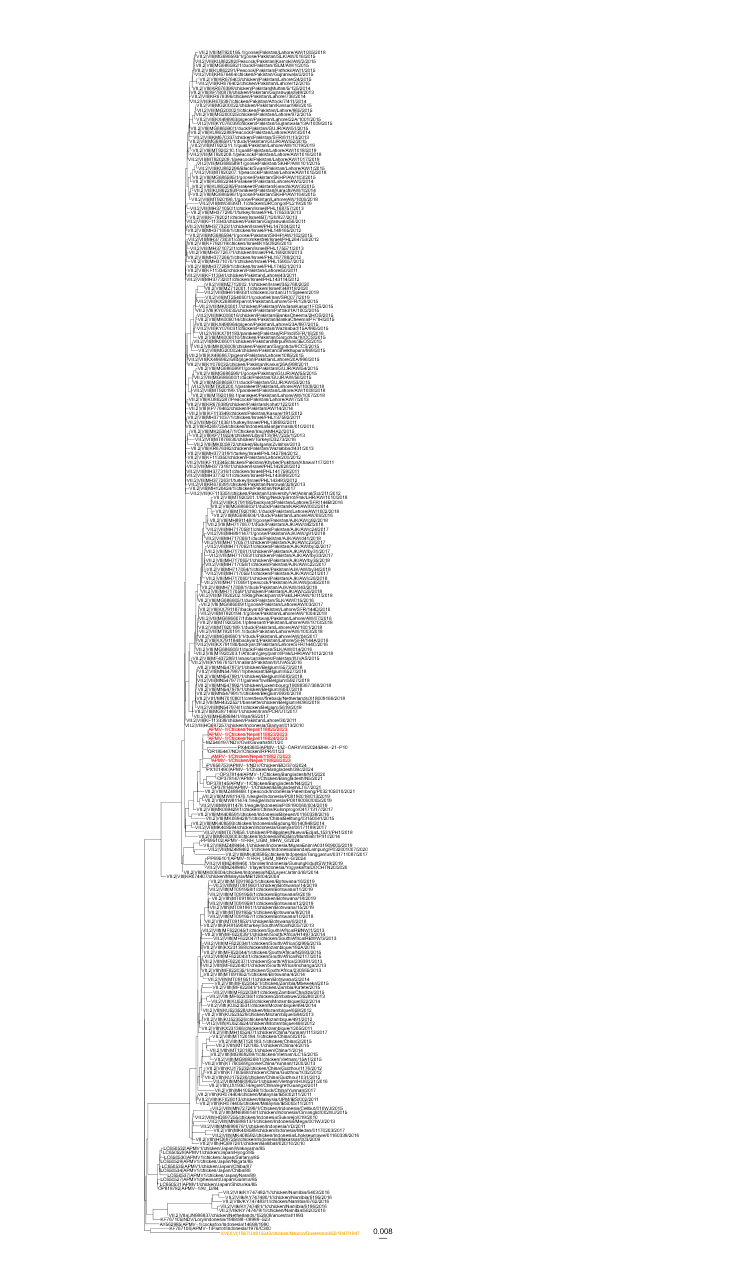
**

**Supplementary figure 2.** Maximum-likelihood analysis of APMV-1 sequences obtained from Nepal genotype VII.2 sequences


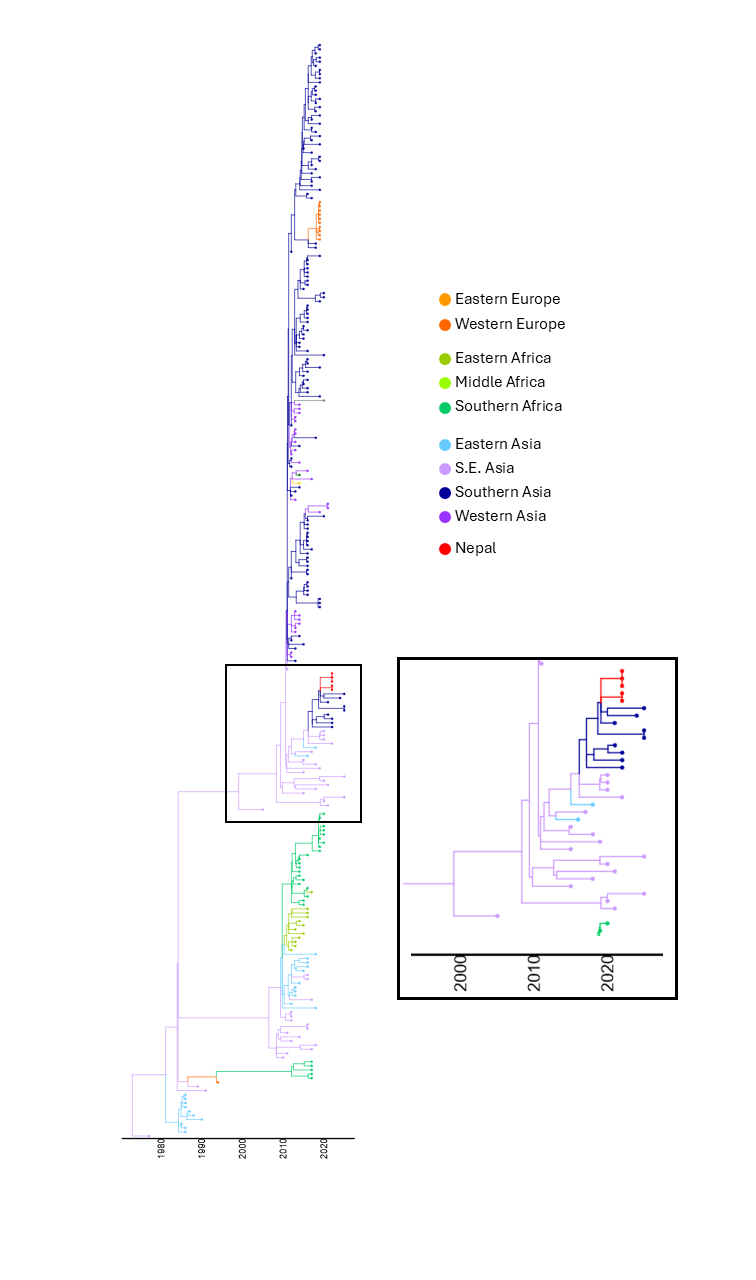


**Supplementary figure 3.** APMV-1 sequences designated genotype VII.2 are closely related to previous strains detected in Southern Asia.

**Table S1: Bayesian Analysis**

|  | **PS** | **SS** |
| --- | --- | --- |
| Relaxed Constant Population | -15524.0728868146 | -15538.372012795 |
| Relax Bayesian SkyRide | -15597.5333797471 | -15616.4331827995 |
| Strict Constant Population | -15660.2475826918 | -15675.2851739637 |
| Strict Bayesian SkyRide | -15705.9235473661 | -15726.4804458805 |
